# Natural killer cell‐related anti‐tumour adoptive cell immunotherapy

**DOI:** 10.1111/jcmm.18362

**Published:** 2024-06-05

**Authors:** Yuwen Qi, Ying Li, Hua Wang, Anjin Wang, Xuelian Liu, Ziyan Liang, Yang Gao, Liqing Wei

**Affiliations:** ^1^ Department of Gynecological Oncology Zhongnan Hospital of Wuhan University Wuhan China; ^2^ Hubei Key Laboratory of Tumor Biological Behaviors Wuhan China; ^3^ Hubei Cancer Clinical Study Center Wuhan China; ^4^ Physical Examination Center Renmin Hospital of Wuhan University Wuhan China; ^5^ Wuhan Wuchang Hospital Wuhan University of Science and Technology Wuhan China

**Keywords:** challenges, chimeric antigen receptor (CAR)‐T cell, immunotherapy, natural killer (NK) cells

## Abstract

Chimeric antigen receptor‐ (CAR‐)modified T cells have been successfully used to treat blood cancer. With the improved research on anti‐tumour adoptive cell therapy, researchers have focused on immune cells other than T lymphocytes. Natural killer (NK) cells have received widespread attention as barriers to natural immunity. Compared to T lymphocyte‐related adoptive cell therapy, the use of NK cells to treat tumours does not cause graft‐versus‐host disease, significantly improving immunity. Moreover, NK cells have more sources than T cells, and the related modified cells are less expensive. NK cells function through several pathways in anti‐tumour mechanisms. Currently, many anti‐tumour clinical trials have used NK cell‐related adoptive cell therapies. In this review, we have summarized the recent progress in NK cell‐related adoptive cellular immunotherapy for tumour treatment and propose the current challenges faced by CAR‐NK cell therapy.

## 1 INTRODUCTION

The recent development of immune checkpoints, represented by programmed cell death protein 1 (PD‐1)/PD‐ligand 1 (PD‐L1) and cytotoxic T‐lymphocyte‐associated protein 4 (CTLA‐4), has revived interest in tumour immunotherapy.^1^ However, immune checkpoint‐based therapies often lead to treatment resistance, resulting in limited therapeutic efficacy.^2^ As scientists delve into the treatment of immune‐related tumours, therapies based on modified immune cells have gradually attracted interest.^3^ To date, six chimeric antigen receptor (CAR)‐T‐cell products have been approved by the US Food and Drug Administration (FDA) for B‐cell leukaemia/lymphoma and multiple myeloma: Kymriah (tisagenlecleucel), Yescarta (axicabtagene ciloleucel), Tecartus (brexucabtagene aut oleucel), Breyanzi (lisocabtagene maraleucel), Abecma (idecabtagene vicleucel) and Carvykti (ciltacabtagene autoleucel).^4^ However, T‐cell engineering‐based therapies have limitations such as the risk of serious toxicity, including cytokine release syndrome (CRS), immune effector cell‐associated neurotoxicity syndrome (ICANS) and graft‐versus‐host disease (GVHD) from allogeneic CAR‐T‐cell therapy.^5^ Therefore, several studies have focused on other immune cells. Natural killer (NK) cells are important innate lymphocytes that have gained popularity.^6^, ^7^ Owing to the advances in our understanding of NK cells and the development of related cell modification strategies, preclinical and clinical research on using NK‐related immune cell therapies for tumour treatment have considerably grown.^8^ Among these, the CAR‐NK therapy has attracted the most attention. This article introduces the recent progress in NK‐related immune cell therapy from the aspects of NK cell characteristics, NK cell sources, NK cell‐related immunotherapy optimization and the challenges faced by CAR‐NK cells.

## 2 NK CELL

NK cells are lymphocytes that play an important role in innate immunity.^9^ NK cells can directly kill virus‐infected or tumour cells without pre‐stimulation or antigen activation; therefore, they are called NK cells.^10^ NK cells differ from common lymphoid progenitor cells and have a T‐ and B‐cell‐independent developmental pathway. Immature CD56^bright^NK cells generally develop into mature CD56^dim^NK cells in the human bone marrow.^11^ This difference in CD56 expression divides NK cells into two subtypes: CD56^bright^NK and CD56^dim^NK cells, which are mainly distributed in the secondary lymphoid organs and peripheral blood, respectively. CD56^bright^NK cells secrete various cytokines, regulate immune responses and possess killing functions.^12^ CD56^dim^NK cells, a terminally differentiated subpopulation, possess a powerful killing ability. The NK cell surface is regulated by the differential expression of activating and inhibitory receptors. During the maturation of CD56^bright^NK cells to CD56^dim^NK cells, the expression of the activating receptor CD16 (also known as FcγRIII) increases, mediating antibody‐dependent cellular cytotoxicity (ADCC).^13^ NK cells respond quickly without pre‐sensitization to antigens and exert killing effects without specificity. This process does not require antibody involvement and is not restricted to the major histocompatibility complex (MHC). Thus, NK cells are the ‘sentinel’ of human immunity.

**FIGURE 1:**
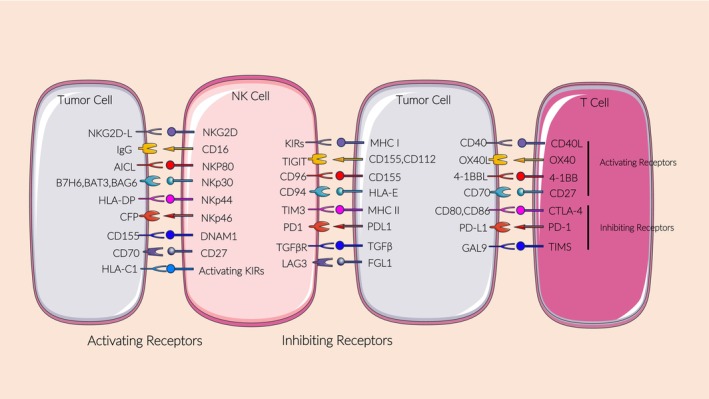
Activating and inhibitory receptors for NK cells. The function of NK cells is determined by their receptor balance. The binding of activating receptors to tumour cell surface receptors mediates the activation of NK cells and exerts cytotoxic effects. Tumour cells express inhibitory receptors that mediate immune evasion. This diagram shows the major activating and inhibitory receptors and their ligands on NK cells.

The response of NK cells to tumours is mainly due to the balance between surface‐activating and ‐inhibitory receptors.^14^ CD16 is the main activating receptor that independently activates NK cells in the absence of cytokine co‐stimulation.^15^ In addition, the activating receptors including NKp30, NKp44, NKp46, NKG2D and 41BB work together to activate the cytotoxic effect of NK cells. Inhibitory receptors on the NK cell surface mainly include killer immunoglobulin‐like receptors (KIR) that bind to MHC class I molecules (mainly human leukocyte antigen [HLA]‐A/B/C). When tumours occur, the level of self‐MHC class I molecules on the target cell surface is downregulated, thus weakening the inhibitory signals and inducing NK cell activation (‘lost self’ mode).^16^ NK cells are usually dormant in blood. However, in the presence of tumours, NK cells are activated and enter the tumour microenvironment to exert their anti‐tumour effects (Figure 1).

The activated NK cells accomplish their killing function through five pathways^17^: (1) promoting tumour cell apoptosis by secreting cytotoxic granules, such as control proteins and granzymes; (2) binding of the activating receptor FcγRIII to the Fc region of IgG to mediate ADCC; (3) releasing soluble NK cytotoxic factor (NKCF), which binds to target cell surface receptors and selectively kills and lyses target cells; (4) inducing target cell apoptosis by expressing membrane tumour necrosis factor (mTNF) family molecules (FASL, TRAIL and mTNF) and binding to target cell membrane ligands; and (5) releasing cytokines (including interferon [IFN]‐γ, TNF, interleukin [IL]‐10, IL‐13 and granulocyte‐macrophage colony‐stimulating factor [GM‐CSF]) that serve as signals for other immune cells to participate in immune responses. These pathways endow NK cells with excellent anti‐tumour capabilities.

Immune memory is a unique feature of adaptive immunity.^18^ NK cells, which are innate immune cells, do not possess an immune memory. However, the adaptive immunity‐like characteristics of NK cells have been discovered recently.^19^ The induction of adaptive NK cells by cytomegalovirus infection is of concern, as it leads to NKG2C^+^ cell expansion.^20^ Non‐specific IL‐12, IL‐15 and IL‐18 activation induces memory‐like NK cell differentiation.^21^ Strategies for using adaptive and memory‐like NK cells in clinical tumour immunotherapy are still being explored.^22^

## 3 NK CELL THERAPY

### 3.1 Advantages of NK cell therapy

Researchers are focusing on NK cell immunotherapy, particularly autologous NK cell therapy, because it is safe.^23^ Consolidation therapy using autologous NK cells has considerable application potential following autologous stem cell transplantation for myeloma. However, the anti‐tumour effects of autologous NK cells are limited by a lack of inhibitory receptors that do not match those of autologous tumour cells. Therefore, allogeneic NK cells have attracted considerable attention. Compared with the current CAR‐T‐cell therapy, allogeneic NK cell therapy has many advantages. First, unlike allogeneic T cells, NK cells do not cause GVHD. Second, unlike T cells, NK cells do not induce CRS or ICANS. CAR‐T cell production typically requires several weeks. However, allogeneic NK cells can be administered immediately. Compared with CAR‐T‐cell therapy, which requires a 1:1 donor, allogeneic NK therapy can use NK cells from one donor for multiple patients. Therefore, it is economically advantageous. As NK cells have multiple anti‐tumour mechanisms, they can effectively reduce immune escape. NK cells can be easily modified using clustered regularly interspaced short palindromic repeats (CRISPR)/Cas9 and base editing technology, and the related products can be optimized in several ways. Researchers are actively exploring the clinical application of allogeneic NK cell therapy.

### 3.2 Different NK cell sources

**FIGURE 2:**
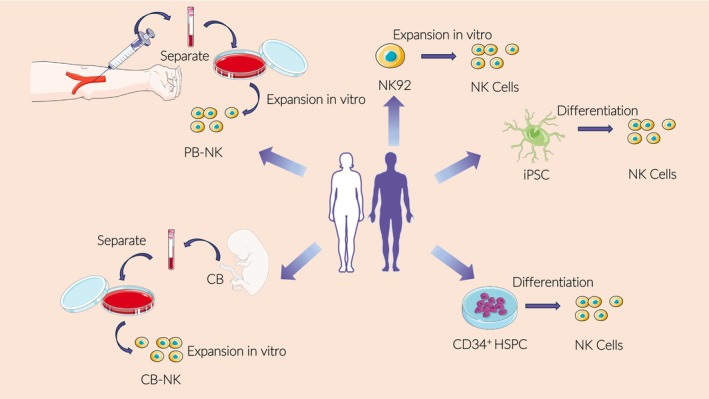
Different NK cell sources. There are five main sources of allogeneic NK cells used for anti‐tumour cell therapy. Peripheral blood is separated and expanded to obtain PB‐NK. NK cell lines are transformed to obtain engineered cells. iPSCs are induced to differentiate to obtain NK cells. CB‐NK cells derived from cord blood. and NK cells induced and differentiated from CD34HSPC. Cells from each source have their own characteristics and advantages. NK cells from different sources have entered clinical trials related to adoptive cell therapy.

NK cells used for therapy are obtained using five methods: peripheral blood‐NK (PB‐NK) cells, NK cell lines, induced pluripotent stem cells (iPSCs), umbilical cord blood (UCB), haematopoietic stem cells and progenitor cells. These pathways produce available NK cells, but different pathways have their own advantages and disadvantages (Table 1), and the source may also potentially impact their function (Figure 2).

**TABLE 1:** Advantages and disadvantages of different cell sources.

NK cells from different sources	Advantages	Disadvantages
PB‐NK	Cells are mature and do not need to undergo a long period of differentiation	The transduction efficiency of PB‐NK is relatively low, and prolonged culture time results in reduced cytotoxicity
NK cell line	Quickly obtain large quantities of cells for treatment; easy to genetically modify	Tumour origin, requiring irradiation
iPSC	Avoid donor heterogeneity and facilitate the development of standardized products	Difficult to proliferate in vivo; potentially immunogenic
CB‐NK	Strong proliferation ability and high production efficiency; the haplotype is easy to determine and can be used to build a cell bank	Different donor sources may lead to product heterogeneity
HSPC‐NK	Conveniently available from blood banks	It requires a longer amplification period than CB‐NK

#### 3.2.1 PB‐NK‐related cell therapy

PB‐NK cells are isolated from the peripheral blood mononuclear cells (PBMCs). As a cellular component isolated directly from the peripheral blood, PB‐NK is simple and easy to obtain. Peripheral blood has mainly mature CD56^dim^NK cells with a strong killing ability. However, PBMCs include only 10%–15% PB‐NK cells. Therefore, researchers have developed various methods to amplify the PB‐NK cells. Although NK cell expansion is only a part of product manufacturing, it affects the safety and effectiveness of the final product.^24^ An ideal NK cell expansion platform must be safe and comply with the Good Manufacturing Practice processes. Additionally, contamination and phenotypic changes during amplification should be avoided. Although NK cells can fully expand, their killing ability must be maintained. NK cell therapy optimization is of interest; therefore, the expansion platform must be compatible with gene editing. We hope that such products can be used in clinical applications; therefore, the amplification platform needs to be fast, inexpensive, and conform to ethical and moral norms.

Current technology for in vitro expanding NK cells relies mainly on feeder cells combined with cytokines.^25^ K562 cells are the most used feeder cell line. K562 is an erythroleukemic cell line that lacks HLA antigen expression. Campana et al. used K562 cells expressing the NK cell‐stimulatory molecule 4‐1BB ligand and IL‐15 to culture PBMCs. In experiments using eight donor samples, NK cells cultured with engineered K562 cells expanded 309‐fold to 12,409‐fold within 3 weeks, and no T‐cell expansion was observed.^26^ NK cells co‐cultured with K562–mb15–41BBL also exhibited anti‐tumour activity in an acute myeloid leukaemia (AML) mouse model. A clinical‐grade amplification process has been developed.^27^ The clinical application of K562–mb15–41BBL remains controversial, and some clinical studies have reported acute GVHD in patients receiving treatment.^28^ Denman et al. developed K562‐based artificial antigen presenting cells (aAPCs) with membrane‐bound IL‐21 (mbIL21), which supported the log phase expansion of NK cells in a 6‐week culture period. NK cells from 22 donors expanded by an average of 47,967‐fold after co‐culturing with mbIL21‐expressing K562 cells for 21 days. Compared with freshly isolated NK cells, expanded NK cells have increased telomere length and do not exhibit cellular senescence.^29^ Phase I clinical trials have proven that the NK cells obtained from this expansion platform are safe. Twenty‐one patients with refractory or beyond first remission AML, myelodysplastic syndrome (MDS) or chronic myelogenous leukaemia received NK cell infusion, demonstrating the good safety profile of allogeneic NK cell therapy.^30^ In addition to K562, a novel NK feeder cell platform was developed using mbIL‐21‐transduced OCI‐AML3 cells (NKF), a platform that induces robust and sustained proliferation of highly cytotoxic NK cells (expansion at 5 weeks by >10,000 times). Upon IL‐2 activation, the expanded NK cells exhibited robust killing of tumour cells. Thus, NKF is a potential new NK cell expansion platform.^31^

Irradiated PBMC can also serve as feeder cells to expand NK cells. Early studies have shown that the use of PBMCs as feeder cells can substantially increase the expansion efficiency of IL‐2‐stimulated NK cells.^32^ Using autologous PBMC as feeder cells theoretically avoids the infusion of malignant feeder cells into the body, but has complicated preparation and long cycles. Other researchers have used irradiated Epstein–Barr virus‐transformed lymphoblastoid cells to expand NK cells. Pure clinical‐grade NK cell populations expanded at least 260‐fold over 21 days. Compared to resting NK cells, expanded NK cells showed increased TRAIL, FasL, and NKG2D expression and were significantly more cytotoxic to bortezomib‐treated tumours.^33^ Freezing decreases NK cell cytotoxicity and NKG2D and TRAIL expression in expanded NK cells. However, this change was reversed upon exposure to IL‐2.

The use of feeder cells during NK cell expansion may pose regulatory challenges and introduce heterogeneity. Although the expansion effect of feeder cell platforms is remarkable, scientists are still attempting to use other expansion systems that do not include feeder cells, such as stimulating granules, nicotinamide and cytokines.^34^, ^35^

#### 3.2.2 NK cell line‐related cell therapy

NK‐92 is an immortalized IL‐2‐dependent lymphoma cell line which was originally isolated from a 50‐year‐old male patient with rapidly progressive non‐Hodgkin's lymphoma.^36^ NK‐92 cells are beneficial because they lack KIR expression and prevent NK cell inhibition. However, NK‐92 cells also lack CD16 expression and inherently cannot kill cells through the ADCC pathway. NK‐92 cells overexpress perforin and granzyme B, thus they are highly cytotoxic and have sufficient potential for tumour immunotherapy.^37^ Extensive research has been conducted on NK‐92 cell‐based anti‐tumour cell therapies. Early studies have shown that NK‐92 is highly cytotoxic to leukocyte cell lines and safe in mouse models.^38^ NK‐92 cells also remove malignant contaminants after autologous stem cell transplantation. Studies have shown that residual chronic myelogenous leukaemia (CML) cells are highly sensitive to clearance by NK‐92 cells. No adverse effects on haematopoietic stem cells were observed.^39^ In 2001, Seifried et al. first reported NK‐92 cell use in clinical trials. This Phase I/II clinical trial involved repeated irradiated NK‐92 cell infusions to eight patients with advanced disease. However, the trial did not yield convincing results because the patients were extremely ill.^40^ Twelve years later, Tonn et al. published the results of a Phase I clinical trial involving 15 patients with advanced refractory malignancies. All patients received two NK‐92 cell infusions, separated by 48 h. The results showed that NK‐92 cells were safe and no serious adverse reactions occurred in any patient. Anti‐tumour responses have been observed in three‐quarters patients with lung cancer.^41^ Owing to the limitations in sample size and trial design, decisions regarding efficacy require further proof in Phase II/III clinical trials. In another phase I clinical trial of NK‐92 cell therapy, researchers administered three NK‐92 cell infusions to 11 patients with refractory metastatic renal cell carcinoma and 1 patient with refractory metastatic melanoma. The results showed that NK‐92 cell cytotoxicity was mild, with only one transient case of Grade 3 fever and one case of Grade 4 hypoglycaemia. Moreover, one patient with refractory metastatic renal cell carcinoma was alive 4 years after infusion. One patient with refractory metastatic melanoma showed mild response to treatment.^42^ Phase I clinical trials have demonstrated the positive effects of NK‐92 cell treatment, and future multicentre randomized controlled clinical trials may provide more convincing evidence. Overall, NK‐92 cells pose a potential risk for infusion because of their tumour origin. Although the safety of NK‐92‐derived NK cells has improved after irradiation, their duration in the body is reduced. NK‐92 is the only NK cell line that has entered immunotherapy trials, carries no risk of GVHD and adapts to various tumours. In addition to NK‐92 cells, KHYG‐1 and YT cells have therapeutic potential.^43^

#### 3.2.3 iPSC‐derived NK cell‐related therapies

In 1981, embryonic pluripotent cell lines were first isolated from in vitro mouse blastocyst cultures.^44^ Researchers are beginning to realize the huge differentiation potential of this cell type. Differentiated cells could be reprogrammed to an embryonic‐like state by transferring nuclear content into oocytes or fusion with embryonic stem (ES) cells. To create cells with high differentiation potential, researchers have induced pluripotent stem cells from mouse embryonic or adult fibroblasts by introducing four factors: Oct3/4, Sox2, c‐Myc and Klf4, under ES cell culture conditions.^45^ In 2007, researchers used four factors (OCT4, SOX2, NANOG and LIN28) to reprogram human somatic cells into pluripotent stem cells that exhibited the basic characteristics of ES cells.^46^ This pluripotent stem cell has the developmental potential to differentiate into the three primary germ layers. iPSCs have become a key tool for drug development, disease models, and developmental research. iPSC‐derived NK cells also show great prospects in anti‐tumour immunotherapy. Due to their high in vitro differentiation potential, iPSCs can produce several homogeneous NK cells. Moreover, during the differentiation process, it is convenient for engineering editing; therefore, it has various optimization characteristics. For example, some researchers introduced CD16 into iPSC so that they have obvious ADCC‐mediating ability after differentiating into NK cells.^47^ Researchers have also performed three‐gene editing in iPSCs. Clonal iPSC lines were engineered to express the high‐affinity, non‐cleavable Fc receptor CD16a and membrane‐bound IL‐15/IL‐15R fusion protein. The third edit was to knockout the extracellular enzyme CD38. The NK cells derived from these uniformly engineered iPSCs display metabolic and gene expression profiles similar to those of cytomegalovirus‐induced adaptive NK cells, persist in vivo in the absence of exogenous cytokines and elicit excellent anti‐tumour activity.^48^ Owing to the high genetic engineering compatibility of iPSC‐derived NK cells, many edited anti‐tumour NK cells have been developed and have entered Phase I clinical trials. In preliminary clinical trials, these iPSC‐NK cells showed acceptable safety; however, their effectiveness awaits further clinical trials.

#### 3.2.4 Cord blood‐derived NK (CB‐NK) cell‐related therapies

Primary NK cells can also be isolated from the UCB. Compared with PB‐NK, which requires a donor, CB‐NK can be obtained more easily from blood banks and has a higher proliferative potential. Although the proportion of NK cells in the UCB is higher than that in the peripheral blood, it needs to be expanded in vitro to reach a clinical level. The in vitro amplification platform was similar to that of PB‐NK, and some teams have used K562/mbIL‐21/41BBL for clinical‐grade amplification.^49^ As CB‐NK comes from a heterogeneous body, it may exhibit inter‐donor variability, which is not conducive to homogeneous mass production.^50^ Compared to PB‐NK, CB‐NK have lower activating receptor levels and higher inhibitory receptor levels, but both showed similar cytotoxicity.^51^ CB‐NK‐related adoptive cellular immunotherapy has also entered clinical trials. In a pivotal Phase I/II trial of the MD Anderson Cancer Centre, 11 patients with relapsed or refractory CD19‐positive cancer (non‐Hodgkin's lymphoma or chronic lymphocytic leukaemia) were injected with UCB‐derived HLA‐mismatched anti‐CD19 CAR‐NK cells. CAR‐NK cells showed a good safety profile. Administration of CAR‐NK cells were not associated with the development of cytokine release syndrome, neurotoxicity or graft‐versus‐host disease, and levels of inflammatory cytokines, including interleukin 6, were not elevated above baseline. Among the 11 treated patients, 8 showed clinical remission.^49^, ^52^ These results are encouraging. Another Phase I clinical trial of CB‐NK cell therapy demonstrated good safety. Twelve patients with multiple myeloma treated with CB‐NK cells did not develop GVHD. Ten patients achieved a remission. CB‐NK was amplified using the mbIL21/K562 platform.^53^ Currently, research on CB‐NK cells is very active, and many related clinical trials have been launched, most of which have focused on CAR‐NK cells based on CB isolation and modification.

#### 3.2.5 NK cell‐related tumour immunotherapy derived from progenitor and haematopoietic stem cells

CD34+ haematopoietic stem and progenitor cells (HSPCs) in UCB can also differentiate and expand into NK cells. Similar to direct isolation from UCB, HSPC‐NK can be easily obtained from blood banks and exhibit inter‐donor variability. Compared with CB‐NK, HSPC‐NK required a longer expansion period. As differentiated HSPC‐NK cells lack CD16 expression, they cannot mediate cytotoxicity through ADCC.^54^ In a first‐in‐human clinical trial, 10 older patients with AML received escalating doses of HSPC‐NK cells without cytokine‐intensified lymphodepleting chemotherapy. The results showed that HSPC‐NK cells were well tolerated, and no GVHD or toxicity was observed.^55^ HSPC‐NK is relatively less developed and lacks tumour‐specific modifications; therefore, it has good prospects for development.

## 4 STRATEGIES TO ENHANCE NK CELL ANTI‐TUMOUR IMMUNOTHERAPY RESPONSE: CAR‐NK CELL THERAPY

### 4.1 CAR‐NK design

CARs usually consist of three parts: an ectodomain, a transmembrane region, and a cytoplasmic activation tail. The most common recognition moieties are single‐chain variable fragments (scFv) of antibodies designed to bind to tumour antigens. CAR‐T cells are currently approved by the FDA for treatment. The design strategy for CAR‐T cells has been used for CAR‐NK cells. Compared with CAR‐T cells, CAR‐NK cells have many potential advantages (Table 2) as they can be readily obtained from various sources, are inexpensive, have rich targeted cell‐killing mechanisms (including intrinsic cytolytic activity and ADCC effects), do not induce GVHD and are safe. The success of Phase I/II clinical trials of CB CD19 CAR‐NK cells has encouraged researchers worldwide, and various developmental strategies have been applied to CAR‐NK cells. In 2023, several clinical trials have been conducted worldwide to test the hypothesis, mainly in China and the United States (Table 3).

**TABLE 2:** Advantages of CAR‐NK cell therapy over CAR‐T‐cell therapy.

	CAR‐T‐cell therapy	CAR‐NK cell therapy
GVHD	High risk	Low risk
CRS	High risk	Low risk
Neurotoxicity	High risk	Low risk
Preparation time	Weeks	Less time
Anit‐tumour mechanism	Antigen killing	Antigen killing, ADCC, intrinsic cytolytic activity
Donor	1:1	Fewer donors
Cost	Higher	Lower

**TABLE 3:** CAR‐NK cells in clinical trials since 2023.

NCT number	Phase	Target	NK source	Condition	Location	Combined therapy	Study start	Status
NCT06045091	Early phase I	BCMA	Unknown	Multiple myeloma, plasma cell leukaemia	Shanghai, China	No	2023/7/4	Recruiting
NCT05776355	Not applicable	NKG2D	Unknown	Ovarian cancer	Hangzhou, Zhejiang, China	No	2023/03	Recruiting
NCT05734898	Not applicable	NKG2D	Unknown	AML	Hangzhou, Zhejiang, China	No	2023/03	Recruiting
NCT05673447	Early phase I	CD19	Unknown	Diffuse large B‐cell lymphoma	Shanghai, China	Fludarabine, cyclophosphamide	2023/3/1	Recruiting
NCT05987696	Phase I	CD33/CLL1	iPSC	AML, AML minimal residual disease	Tianjin, China	Cyclophosphamid, fludarabine, cytarabine	2023/8/10	Not yet recruiting
NCT05739227	Early phase I	CD19	PB‐NK	Acute lymphoblastic leukaemia, B‐cell lymphoma, chronic lymphocytic leukaemia	Xuzhou, Jiangsu, China	Fludarabine, cyclophosphamide and etoposide	2023/3/1	Recruiting
NCT06027853	Phase I	CLL1	iPSC	AML	Hangzhou, Zhejiang, China	Cyclophosphamid, fludarabine, VP‐16	2023/9/10	Recruiting
NCT06006403	Phase 1/Phase 2	CD123	Unknown	Acute myeloid leukaemia, blastic plasmacytoid dendritic cell neoplasm (BPDCN), refractory leukaemia, relapse leukaemia	Taiyuan, Shanxi, China	Fludarabine, cyclophosphamide	2023/8/31	Recruiting
NCT06010472	Early phase 1	CD19	Unknown	Systemic lupus erythematosus (SLE)	Shanghai, China	Fludarabine, cyclophosphamide	2023/8/24	Recruiting
NCT05845502	Not applicable	Unknown	Unknown	Advanced hepatocellular carcinoma	Unknown	No	2023/5/4	Not yet recruiting
NCT05922930	Phase I/II	TROP2	CB‐NK	Adenocarcinoma, ovarian cancer, pancreatic cancer	Houston, Texas, United States	Cyclophosphamide, fludarabine	2023/12/31	Not yet recruiting
NCT06066424	Phase 1	TROP2	CB‐NK	Solid TUMOURS	Houston, Texas, United States	Rimiducid, fludarabine phosphate, cyclophosphamide	2023/10/24	Recruiting
NCT05110742	Phase I/II	CD5	CB‐NK	Haematological malignancy	Houston, Texas, United States	Fludarabine phosphate, cyclophosphamide	2023/11/30	Not yet recruiting
NCT05703854	Phase I/II	CD70	CB‐NK	Advanced mesothelioma, advanced osteosarcoma, advanced renal cell carcinoma	Houston, Texas, United States	Fludarabine phosphate, cyclophosphamide	2023/3/29	Recruiting
NCT05856643	Early phase I	Unknown	Unknown	Ovarian epithelial carcinoma	Unknown	No	2023/6/1	Not yet recruiting
NCT05842707	Phase I/II	CD19/CD70	CB‐NK	Refractory or relapsed B‐cell non‐Hodgkin lymphoma	Shanghai, Shanghai, China	No	2023/1/18	Recruiting
NCT05700630	Phase I	CD16	iPSC	HIV‐1‐infection, ART, Cd4+ lymphocyte deficiency, lymphoid tissue; infection, interleukin	Minneapolis, Minnesota, United States	Vorinostat	2024/7/15	Not yet recruiting
NCT05336409	Phase I	CD19	iPSC	R/R CD19‐positive B‐cell malignancies, indolent non‐Hodgkin lymphoma, aggressive non‐Hodgkin lymphoma	Multi‐centre in United States	Lymphodepleting chemotherapy, IL‐2	2023/1/24	Recruiting
NCT05618925	Phase I	CD19	NK‐92	Non‐Hodgkin's lymphoma refractory/relapsed	El Segundo, California, United States	IL‐15, cyclophosphamide, fludarabine, rituximab	2023/11/1	Not yet recruiting

**FIGURE 3:**
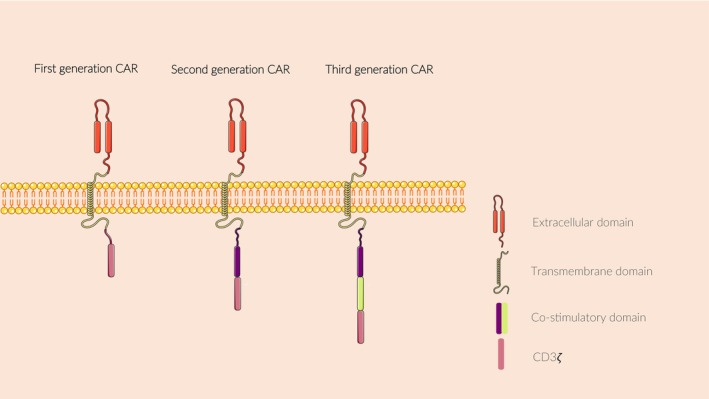
CAR structure. The structure of CAR is mainly composed of extracellular domain, transmembrane domain and intracellular domain. The main difference between different generations of CAR is the co‐stimulatory binding domain.

In addition to the design experience based on CAR‐T cells, researchers have focused on NK cell‐related autostimulatory signals. Temme et al. designed a DNAX activator protein 12 (DAP12)‐based CAR‐targeting prostate stem cell model.^56^ DAP12 is a signal transduction molecule involved in activating NK cell receptor signal transduction.^57^ DAP12 activates NK cells by interacting with KIR family molecules. The number of intracellular CAR activation signals defines its ‘generation’. The first‐generation CAR has only one intracellular activation signal, and the second‐generation has two (Figure 3). Currently, second‐generation CAR costimulatory signals commonly used in NK cells include CD3ze, CD28, the TNF receptor (TNFR) gene family (including 4‐1BB, OX40 and CD27) and signalling lymphocyte‐activating molecule (SLAM)‐related receptor family (including the 2B4).^54^, ^58^ In the CAR‐NK tumour cell recognition region, the most common target in blood cancers and solid tumours are CD19 and HER2, respectively.

### 4.2 CAR‐NK optimization strategy

#### 4.2.1 Design‐based optimization strategies

Currently, strategies to improve CAR‐NK cell therapy mainly focus on avoiding antigen escape, increasing activation control mechanisms and recognizing intracellular signals. The major challenge in CAR‐based cellular immunotherapy is the prevention of tumour antigen escape. Researchers have attempted to address this issue by targeting multiple antigens. Using bispecific CARs, generally second‐/third‐generation CARs, a single or double antibody‐binding activation mode can be designed for accurate tumour antigen recognition. Currently, research on bispecific CARs mainly focuses on CAR‐T therapy. However, it is expected to have broad applications.

To avoid NK cell activation by non‐tumour recognition, a gating switch was also designed. The researchers used a proprietary bioinformatics paired antigen discovery platform to identify the optimal combination of AML tumour‐associated and healthy tissue antigens and then designed OR and NOT logic‐gated CARs to control activation signals. The NOT gate is based on targets expressed in normal tissues, and acts as a protective agent. Other researchers have chosen to protect HSCs from injury using VSIG2, CPM and SLC26A2.^59^

Generally, the recognition ability of the CAR is limited to tumour cell surface proteins. However, capturing intracellular antigens is difficult. Researchers have designed T‐cell receptor (TCR)‐expressing NK‐92‐based NK cells. TCRs recognize antigenic peptides in degraded proteins present in MHC molecules. Therefore, TCR recognition was not affected by the subcellular protein localization. Modified NK cells have an expanded recognition range and are a feasible solution.^60^

#### 4.2.2 Delivery of NK cells to tumours

Insufficient immune cell infiltration in solid tumours is a key limitation of cellular immunotherapies. NK cell delivery to tumour sites is an important factor in improving anti‐tumour efficiency. Chemokine expression is a ‘guide’ for immune cells. Enhancing the expression of NK cell chemokine receptors enhance their trafficking to the tumour area. Cell engineering has been used to stably express CXCR1, CXCR2, CXCR3, CXCR4 and other chemokine receptors to improve the anti‐tumour efficacy of CAR‐NK cells.^61^, ^62^, ^63^, ^64^, ^65^ Chemokines released by tumours may need to be artificially increased to attract NK cells for homing, and a strong heterogeneity may be present among different tumour cells, which requires a personalized decision to promote NK cell homing. These questions should be addressed in future studies.

#### 4.2.3 Cytokine armoured CAR‐NK

NK cells are activated by stimulation with cytokines (including IL‐2, IL‐15 and IL‐21). When CAR‐NK cells exert anti‐tumour effects, cytokine stimulation enables them to exert better cytotoxicity. IL‐12, IL‐15 and IL‐18 can induce memory‐like NK cells, and using cytokines to pre‐activate memory‐like NK cells may improve anti‐tumour efficiency. However, prolonged NK cell stimulation by cytokines may cause fatigue.^66^ Therefore, cell engineering has been used to engineer autocrine NK cells. IL‐15‐armoured CAR‐NK cells are currently being studied and have achieved good preclinical results.^48^

#### 4.2.4 Other optimization strategies

NK cell function is determined by both activating and inhibitory receptors on the cells. For NK cells to exert sufficient cell‐killing power, inhibitory receptors must be blocked. Although antibody‐blocking KIR have failed to achieve ideal clinical trial results, recent KIR‐based designs of CAR‐NK have overcome phagocytosis‐mediated cannibalism and tumour escape.^67^ Many immune checkpoints (including TIM‐3, TIGIT, LAG3, PD‐1, CD161, and CD96) are also NK cell inhibitory receptors, and the combination of immune checkpoint inhibitors or the development of CAR‐NK based on these checkpoints seems to increase anti‐tumour efficacy. NK cells target tumour cells via their adapters. In addition to bispecific binders that directly cross link tumour and NK cells, tri‐ or tetra‐specific binders targeting multiple antigens are also being developed. Studies have shown that by using a tri‐specific molecule targeting CLEC12A on AML cells and activating NK cells with a humanized anti‐CD16 single‐domain antibody and IL‐15, the NK cell‐mediated response has a strong effect on primary patient‐derived AML blasts.

### 4.3 CAR‐NK in clinical trials

There are currently a large number of clinical trials focusing on CAR‐NK therapy. Some early clinical trials have been completed with positive results as well. In a Phase I/II clinical trial, researchers used CAR‐NK to treat patients with refractory or relapsed CD19^+^ lymphoma. The results showed that among the 11 patients treated, no major toxic effects were observed, and eight patients experienced remission.^52^ In another study, researchers used umbilical cord blood‐derived CAR‐NK cells to treat patients with CD19^+^ B‐cell tumours. The results showed that among 37 patients, no obvious toxicity was observed. The response rates on both Day 30 and Day 100 were 48.6%. Interestingly, the researchers observed that patients who achieved OR had higher levels of CAR‐NK cells.^68^

## 5 CHALLENGES FACED BY CAR‐NK

Although CAR‐NK has achieved positive results in Phase I clinical trials, it still faces many challenges in clinical anti‐tumour applications. Owing to the possible risks of allogeneic cells, new expansion platforms need to be developed in the future to reduce dependence on feeder cells. Owing to the difficulty and cost of clinical application, the NK cell freezing process should be optimized and their production cycle should be shortened. However, the application of CAR‐NK cells to solid tumours still faces many challenges. The complex tumour microenvironment and limited infiltration affect the killing effect of CAR‐NK cells. The killing effect has been improved after knocking out CISH in NK cells, activating the mTOR pathway, and improving tumour metabolism and the microenvironment. However, further validation through clinical trials is required. The current CAR is designed for constructing CAR‐T cells, so it may not be the best choice for NK cells. The location of the CAR‐binding epitope and its distance from the surface of CAR‐NK cells affect its ability to bind antigen and activate CAR‐NK cells. Therefore, it is particularly important to design new CAR structures for NK cells. The difficulty in transduction or the risk of T‐cell contamination caused by the selection of NK cell sources will also limit the clinical application of CAR‐NK. There is currently a lack of regulatory compliance regarding the production of NK cells, which may pose risks. Although CAR‐NK faces many challenges as an anti‐tumour therapy, it is a promising treatment. In the future, we look forward to the development of safe and effective CAR‐NK cells and their implementation in clinical practice.

## AUTHOR CONTRIBUTIONS

**Yuwen Qi:** Investigation (equal); methodology (equal); writing – original draft (equal). **Ying Li:** Methodology (equal); writing – original draft (equal). **Hua Wang:** Writing – review and editing (equal). **Anjin Wang:** Investigation (supporting). **Xuelian Liu:** Investigation (supporting). **Ziyan Liang:** Investigation (supporting). **Yang Gao:** Conceptualization (equal); project administration (equal); writing – review and editing (equal). **Liqing Wei:** Conceptualization (equal); project administration (equal).

## FUNDING INFORMATION

This work was supported by supported by ‘the Fundamental Research Funds for the Central Universities (Project:2042023kf0056)’ and Zhongnan Hospital of Wuhan University Science, Technology and Innovation Seed Fund (Project: CXPY2022024).

## CONFLICT OF INTEREST STATEMENT

The authors confirm that there are no conflicts of interest.

## Data Availability

Data availability is not applicable to this article as no new data were created or analyzed in this study.
